# The prevalence of *Dichelobacter nodosus* in clinically footrot-free sheep flocks: a comparative field study on elimination strategies

**DOI:** 10.1186/s12917-020-2243-8

**Published:** 2020-01-22

**Authors:** A. F. Kraft, H. Strobel, J. Hilke, A. Steiner, P. Kuhnert

**Affiliations:** 1 Department of Clinical Veterinary Medicine, Clinic for Ruminants, Vetsuisse-Faculty, University of Bern, Bremgartenstrasse 109a, CH-3001 Bern, Switzerland; 2 Schafpraxis, Am Hopfenberg 8, 89352 Stoffenried, Germany; 3 Institute of Veterinary Bacteriology, Vetsuisse-Faculty, University of Bern, Länggassstrasse 122, CH-3001 Bern, Switzerland

**Keywords:** Footrot, Lameness, Sheep, Foot, *Dichelobacter nodosus*, *aprV2*, *aprB2*, Elimination program

## Abstract

**Background:**

Ovine footrot caused by *Dichelobacter nodosus* (*D nodosus*) is an infectious disease affecting sheep worldwide. Switzerland plans a nationwide footrot eradication program, based on PCR-testing of interdigital swab samples. The aim of this study was to test for the presence of *D nodosus* in clinically footrot-free sheep flocks which had been subjected to different treatment strategies, to assess whether they were feasible for the eradication process, especially focussing on antimicrobial flock treatments. Clinical scoring and PCR-results were compared. Ten farms had used hoof bathing and hoof trimming without causing bleeding, ten had used individual treatments and flock vaccines to gain the free status and ten had become free through whole-flock systemic macrolide treatment. For every farm, three risk-based collected pool samples were analysed for the occurrence of virulent and benign *D nodosus* by PCR detection of *aprV2*/*aprB2*.

**Results:**

Six flocks from any treatment group tested positive for *aprB2* in all pools*.* Clinical signs were absent at the time of sampling, but some flocks had experienced non-progressive interdigital inflammation previously. Two flocks tested *aprV2*-positive in the high-risk pool. One of them underwent a progressive footrot outbreak shortly after sampling. Individual retesting indicated, that virulent *D nodosus* most likely was reintroduced by a recently purchased ram. In the second flock, a ram was tested positive and treated before clinical signs occurred.

**Conclusions:**

All treatment strategies eliminated the causative agent and were found to be suitable for implementation in the PCR-based eradication process. PCR-testing proved to be more sensitive than visual scoring, as it also detected clinically healthy carriers. It will be of benefit as a diagnostic tool in elimination and surveillance programs.

## Background

Ovine footrot is a bacterial infection of the interdigital skin of sheep challenging producers worldwide [1]. It is characterised by a strong odour and an exudative inflammation which leads to epidermal necrosis and, in severe cases, separation of the hoof horn capsule from the underlying dermal tissue [2]. This highly painful disease causes severe lameness and is therefore a serious animal welfare issue in sheep-producing countries [3–5]. Furthermore, it leads to financial losses in meat, milk and wool production due to poor ewe fertility, lower lamb growth rates and reduced sales opportunities for infected livestock [4, 6, 7]. The essential causative agent of footrot is *Dichelobacter nodosus* (*D nodosus*), a Gram-negative, anaerobic bacterium which thrives in moist and warm environmental conditions [8, 9].

*D nodosus* isolates can be categorised as either benign or virulent by their expression of the *aprB2* and *aprV2* gene, respectively, which code for thermolabile or thermostable acidic proteases [10]. They are important enzymes in the characteristic tissue-destructive pathogenesis of footrot and are assumed to be the key virulence factors of *D nodosus* [11–13]. Virulent strains lead to severe lameness with the typical underrunning of the hoof horn (clinical footrot), while benign strains tend to cause non-progressive inflammation of the interdigital skin (interdigital dermatitis). In this paper, the term *clinical footrot* is used for severe inflammation which is not restricted to the interdigital space and includes progressive underrunning of the horn capsule (score > 2) contrarily to interdigital dermatitis (score 1–2). For differentiation, the scoring system of the Swiss Consulting and Health Service for Small Ruminants was used [14]. As early stages of footrot cannot be distinguished from other interdigital infections by clinical signs alone [15], the development of appropriate diagnostic tools is important [1]. Stäuble et al. [10] implemented a real-time PCR, which enables early simultaneous detection and discrimination of *aprB2* and *aprV2* positive strains directly from clinical samples.

Footrot is the economically most relevant infectious hoof disease in sheep [16]. Cost-benefit-analyses support elimination programs to obliterate the disease [14, 17, 18]. Different treatment strategies have been assessed all over the world, ranging from total elimination of the causative agent by culling infected flocks to genetic selection of resistant animals and use of specific vaccines [19, 20]. Antimicrobial whole-flock treatments deliver promising results throughout Europe in terms of elimination of clinically apparent footrot [17, 21, 22], but are also criticised regarding the emerging antimicrobial resistance in human and veterinary medicine. Gamithromycin, a macrolide of the azalide subclass, has been licensed for the treatment of footrot in sheep in Germany and other European countries since 2017. It is well suited for antimicrobial elimination of footrot due to clinical cure rates of more than 90% after a single injection [23] and due to its user-friendliness. In addition, it has no known side effects for the animal, concentrates in skin and inflamed tissue and therapeutic concentrations in the hoof are maintained, with a half-life of 5.25 days [24].

The objective of the present study was to find out whether sheep flocks which became clinically free from footrot after a single antimicrobial whole flock treatment with macrolides were also free from virulent *D nodosus* as tested by real-time PCR of pooled swab samples (group C). This was compared with PCR-results from flocks which had been clinically free from footrot for a period of 10 years or more and had used footbathing and hoof trimming without causing bleeding (group A) and to flocks which had become clinically free at least 2 years before sampling, following a protocol including foot-bathing, topical and/or systemic application of antimicrobials and vaccination with a commercial vaccine (group B). Furthermore, the possible benefits of a PCR-based footrot elimination program compared with an elimination protocol based on clinical signs were assessed.

## Results

Seven sheep from three different flocks (three animals on farm 7, two animals on farm 9 and two animals on farm 15) had a footrot score of 1 (mild interdigital inflammation), whilst the rest scored 0. Table 1 shows the PCR-outcome for the pooled flock samples, grouped according to their treatment strategy.

**Table 1:** Pooled sample PCR-results for farms 1–30, grouped according to the chosen treatment method

Group	Farm number	Flock size	Footrot free since	Pool 1–10		Pool 11–20		Pool 21–30	
				aprV2	aprB2	aprV2	aprB2	aprV2	aprB2
A	6	700	before 2008	neg.	neg.	neg.	neg.	neg.	neg.
	13	400		neg.	neg.	neg.	neg.	neg.	neg.
	14	1000		neg.	pos. ct36	neg.	pos. ct26	neg.	pos. ct30
	20	800		neg.	neg.	neg.	neg.	neg.	neg.
	21	250		neg.	pos. ct22	neg.	pos. ct27	neg.	pos. ct27
	26	950		neg.	neg.	neg.	neg.	neg.	neg.
	27	750		neg.	neg.	neg.	neg.	neg.	neg.
	28	150		neg.	neg.	neg.	neg.	neg.	neg.
	29	650		neg.	pos. ct24	neg.	pos. ct30	neg.	pos. ct28
	30	250		neg.	neg.	neg.	neg.	neg.	neg.
B	1	800	2015	neg.	neg.	neg.	neg.	neg.	neg.
	2	2800	2016	neg.	neg.	neg.	neg.	neg.	neg.
	3	800	2014	neg.	neg.	neg.	neg.	neg.	neg.
	5	750	2010	neg.	neg.	neg.	neg.	neg.	neg.
	12	850	2015	neg.	neg.	neg.	neg.	neg.	neg.
	16	550	2013	neg.	neg.	neg.	neg.	neg.	neg.
	17	600	2015	neg.	neg.	neg.	neg.	neg.	neg.
	18	600	2012	neg.	pos. ct27	neg.	pos. ct29	neg.	pos. ct32
	24	120	2013	pos. ct35	neg.	neg.	neg.	neg.	neg.
	25	100	2016	neg.	pos. ct35	neg.	pos. ct24	neg.	pos. ct26
C	4	70	2014	neg.	neg.	neg.	neg.	neg.	neg.
	7	850	2015	neg.	neg.	neg.	neg.	neg.	neg.
	8	650	2014	neg.	neg.	neg.	neg.	neg.	neg.
	9	1100	2015	pos. ct22	pos. ct23	neg.	pos. ct24	neg.	pos. ct24
	10	150	2011	neg.	neg.	neg.	neg.	neg.	neg.
	11	900	2014	neg.	neg.	neg.	neg.	neg.	neg.
	15	800	2014	neg.	neg.	neg.	neg.	neg.	neg.
	19	450	2011	neg.	neg.	neg.	neg.	neg.	neg.
	22	250	2017	neg.	neg.	neg.	neg.	neg.	neg.
	23	400	2017	neg.	neg.	neg.	neg.	neg.	neg.

Only two flocks (farm 9 (group C) and farm 24 (group B)) tested positive for virulent *D nodosus* (*aprV2*-positive), and in both cases, only the high-risk pools were positive. On farm 9, two animals of the positive pool had a footrot score of 1, while the rest of this flock was scored 0, just as all ten sheep in the positive high-risk pool of farm 24 did (Table 2).

**Table 2:** Individual footrot scores and PCR-results of the sheep in the two *aprV2*-positive pool groups

Farm number	Sample number	Sex	Footrot score	aprV2	aprB2
9	9/01	male	1	pos. ct19	neg.
	9/02	male	1	pos. ct28	neg.
	9/03	male	0	pos. ct20	pos. ct26
	9/04	female	0	pos. ct29	pos. ct26
	9/05	female	0	neg.	pos. ct24
	9/06	female	0	pos. ct38	pos. ct27
	9/07	female	0	neg.	pos. ct24
	9/08	female	0	pos. ct36	pos. ct26
	9/09	female	0	neg.	pos. ct26
	9/10	female	0	neg.	pos. ct24
24	24/01	male	0	pos. ct36	neg.
	24/02	female	0	neg.	neg.
	24/03	female	0	neg.	neg.
	24/04	female	0	neg.	neg.
	24/05	female	0	neg.	neg.
	24/06	female	0	neg.	neg.
	24/07	female	0	neg.	neg.
	24/08	female	0	neg.	neg.
	24/09	female	0	neg.	neg.
	24/10	female	0	neg.	neg.

Benign *D nodosus* (*aprB2*-positive) was found in six flocks and always in all three pools. All animals in the positive pools had a footrot score of 0. In group A, three farms tested positive (farm 14, 21 and 29), in group B there were two farms (farm 18 and 25) and in group C only a single farm tested positive (farm 9) for the *aprB2* gene.

Four pools were selected for analysis of single animal samples. Two of these were the high-risk pools of farms 9 and 24, which had been tested positive for virulent *D nodosus*. Table 2 shows the results of the individually tested samples. In pool 1 of farm 9 (group C), several combinations were found: Two animals were only positive for *aprV2*. They had both scored 1 in the clinical footrot score. Four animals were only positive for *aprB2*, and four were positive for both alleles. Those eight animals had scored 0 when clinically examined. When investigating for detailed information about the pool group, it was found, that one of the two only *aprV2*-positive samples with the score 1 was from a recently purchased ram. Two weeks after sampling, the flock had a footrot outbreak with progressive clinical signs.

Pool 1 of farm 24 revealed the following results when single samples were tested: only one animal tested positive for *aprV2*. The other samples were negative for *D nodosus*. The whole group had a score 0 when sampled. Further investigations on the farm resulted in the information, that the positive animal, a ram, was newly purchased and recently introduced into the flock. The ram still scored 0 as well as the rest of the flock at the next veterinary farm visit 18 days later, but the ram was separated from the flock after the positive result was given, and he was treated with gamithromycin as a biosecurity measure and due to animal welfare reasons.

The other retested samples were the high-risk pools of farms 22 and 23 since they showed exceptionally high Ct-values of up to 38 in the internal positive control (IPC) in all pool groups indicative for the presence of PCR inhibitors in the pool samples. However, tested single samples of the high-risk pools confirmed the negative results for these farms.

All farms were regularly revisited by the farm vet (at least once every 3 months). The farms lameness score was assessed at all visits. A regular hoof screening was performed by the farmer at routine hoof trimming. Every sheep and every hoof was inspected. All farms stayed clinically healthy for the observed period of 12 months after the samples were taken, excluding farm 9.

## Discussion

The present study demonstrates that antimicrobial whole-flock treatments, combined with footbathing, can be a useful tool to eliminate both clinical signs of footrot and the causative agent, i.e. virulent *D nodosus*. This makes antimicrobial treatments applicable for elimination programs based on PCR results, always under consideration of the disadvantages of using critically important antimicrobials. Real-time PCR testing proved to be a more sensitive method than clinical examination, as it also identified clinically healthy carriers of *D nodosus*.

Ideally, samples would have been collected before the elimination process as well, however this was not an experimental but a field study. It remains unclear, whether the *aprB2*-positive farms never eliminated benign *D nodosus* in their flocks, due to healthy carriers which never received any treatments, or whether the farmers introduced purchased carriers without clinical signs to the flock which again spread the agent. The latter can be assumed likely in flock 9, as whole flock antimicrobial treatments eliminate both genotypes, and the biosecurity deficits regarding the new outbreak support this hypothesis.

The risk-based pool-of-10 sampling strategy worked well in our setting, since all animals positive for virulent *D nodosus* were found in the high-risk group. Pooling and pool size were found to be reliable and appropriate. This supports the application of risk-based pooling for flock screenings, elimination and surveillance programs, as it is cost and labour effective.

One major challenge in footrot elimination is the development of diagnostic tests which can discriminate reliably between target and non-target strains [1]. Regional elimination programs are usually aimed at virulent *D nodosus*, as large-scale elimination of benign *D nodosus* is not economically justifiable [25]. In Switzerland, the use of the PCR test was found to be the most economic and sensitive way to eliminate footrot in the planned nationwide control program [18]. Moreover, the PCR test can be applied to other ruminants like goats and cattle or camelids, which is important for targeted disease control [26]. In the current study, it detected symptomless carriers of virulent and benign *D nodosus* before clinical signs were manifest and proved thereby to be more sensitive than clinical scoring. The PCR results agreed with the clinical appearance of virulent and benign strains, including subclinical carriers, in agreement with previous observations [10, 27, 28]. Virulent *D nodosus* caused progressive underrunning footrot in flock 9 during the outbreak that followed sampling. When the farmer discovered the reinfection, 20% of the flock was already infected and had to be treated. Two weeks earlier, also the farm of origin of the infected ram had noticed a new footrot outbreak.

In flock 24, no clinical signs were observed when the flock was revisited 3 weeks later. It must be noted that the single positive animal in that flock was again the ram. It had a low load of virulent *D nodosus* based on the high Ct-value and underwent immediate antimicrobial treatment, once the positive result was known.

Likewise, there were no clinical signs in the flocks positive for benign *D nodosus* at the time of sampling, probably due to dry weather conditions. According to the flock health veterinarian, *aprB2*-positive *D nodosus* occasionally caused either mild inflammations (likely in lambs due to increased vulnerability of their interdigital space in rough conditions) or severe inflammations with acute lameness, but no underrunning (H. Strobel, personal communication). It can be concluded that clinical features are consistent with the PCR classification if benign is defined as non-progressive in the South German sheep population. This is in line with findings from Locher et al. [27] in a previous study on clinically free sheep flocks in Switzerland, was described in Australia by Best et al. [29] and also reported from Norwegian farms by Vatn et al. [28]. Further, Best et al. [29] proofed, that real-time PCR was significantly more sensitive in detection of *aprV2* in clinically healthy sheep compared with culture/gelatinase test. McPherson et al. stated, that the results of the real-time PCR did not agree sufficiently with the clinical findings in Australian sheep flocks and therefore depended on additional culturing [30]. In Sweden benign *D nodosus* is often associated with underrunning (score ≥ 3 lesions) [31]. Similarly, clinical impact caused by benign *D nodosus* was reported from a wild-range alpine ibex colony in Switzerland [32]. Therefore, further research is required to determine whether the PCR classification used in this study corresponds to clinical presentation, if considered as elimination tool in other species like goats or other populations, since the genetic background of the host also plays a role [33]. If clinical disease expression and PCR results match each other, the test is a useful diagnostic tool for early diagnosis of virulent *D nodosus* at the flock level and can support veterinarians to distinguish between footrot and interdigital inflammations of other cause at an early stage of disease, when clinical signs are not definite [14]. This will lead to reduced antimicrobial use, as non-progressive interdigital inflammations respond well to alternative treatments.

Animal welfare is an important concern in today’s society, and footrot is one of the main welfare issues in sheep production [34]. Antimicrobial whole-flock treatments have shown successful results for elimination of the disease [22]. Strobel et al. [23] reported cure rates of more than 99% after one or two systemic administrations. Therefore, if a single antimicrobial treatment can improve animal welfare significantly, it should be considered [35]. On the other hand, the World Health Organization considers the popularly used macrolides as “critically important antimicrobials” for human medicine, and gives them highest priority within this category, suggesting to restrict their use if alternative treatments are available [36]. This puts pressure on veterinarians to limit antimicrobial use [37]. Before implementing a footrot elimination program based on antimicrobials, it must be investigated, whether strict biosecurity measures can be fulfilled after the elimination to avoid reinfections, repeated antimicrobial treatments and economic losses. Especially the farmer’s engagement and will to eliminate the disease is mentioned as a key factor for successful elimination [1, 29, 38], besides culling of non-responders and strict quarantine of purchased animals [15, 38]. Our study shows that monitoring the whole flock remains essential even after clinical elimination, and the *aprV2*/*aprB2* PCR is a very useful tool for this purpose.

Whilst macrolide treatments are at present an effective tool to eliminate virulent *D nodosus*, it is likely, that they will no longer be licensed or effective in the future. Repeated footbathing in 10% ZnSO_4_ solution proved to be effective for the elimination of virulent strains of *D nodosus* in a proof-of-concept study [39]. However, since the application of disinfectants based on zinc, copper or formol solution is debatable, further investigation on alternative foot bath solutions and alternative ways to treat clinical footrot is desirable. The reintroduction of virulent or benign *D nodosus* to a sanitized flock through a clinically healthy carrier must be considered the main risk [2], and was also described in a field study by Forbes et al. [22]. Rams were found to be a major risk factor as they often change hands and are being shown in sheep markets. The establishment of a footrot free flock certification, which is based on a negative PCR outcome for *D nodosus,* as Stamphøj et al. [21] suggested, would allow farmers to select rams from footrot free breeders, which might be a key factor for footrot elimination in European sheep production. Ram breeders would be obliged to join in an accreditation scheme.

## Conclusions

In conclusion, this study shows that a single macrolide whole-flock treatment combined with footbathing could eliminate both footrot and its causative agent, virulent *D nodosus*, from an infected flock, as well as repeated footbathing according to the conventional Swiss protocol [39] and selective treatments did.

Our findings implicate that the real-time PCR test will be of benefit as a diagnostic tool in elimination and surveillance programs, as it identifies carriers before clinical signs are manifest. This may reduce the severity of an outbreak, because early intervention is possible, and will lead to improved animal welfare and reduced losses. Additionally, it allows the discrimination between early stages of footrot and interdigital lesions of other origin, which helps to reduce antimicrobial treatments to a minimum.

## Methods

### Study groups

Thirty sheep farms were selected from clients of either Schafpraxis or the Sheep Flock Health Service in Baden-Württemberg in Southern Germany. The farmers had used different strategies to eliminate footrot, and flocks were divided into three groups:

- Group A: Flocks which had no cases of clinical footrot in the past 10 years or longer and did not vaccinate during this period.
- Group B: Flocks which were at least 2 years free from clinical footrot. They had become free through repeated individual treatments with antimicrobials (long-acting oxytetracycline, erythromycin, florfenicol), repeated zinc sulphate footbaths (Golden Hoof plus®, Sheepfair Products) and separation of clinically affected sheep from the flock. Visual whole-flock hoof-screening was operated within 1 day when new interdigital inflammations were observed. Ahead of risky weather periods, the whole flock was immunised with a commercial vaccine (Footvax®, MSD Animal Health) [40].
- Group C: Flocks which became clinically free by a single systemic whole-flock antimicrobial treatment. If an animal did not clinically heal within 21 days, it was treated a second time, non-responders were culled [23]. Seven of the selected flocks had used gamithromycin (Zactran®, Boehringer Ingelheim), two had used erythromycin (Erythrocin vet. 200®, Ceva Animal Health) and one flock had used both products for the elimination process. All flocks of this group proved to have overcome at least three challenging weather conditions without clinical signs of reinfection.

After the elimination of footrot, the only treatment in all three groups were single zinc sulphate footbaths after potential contact with infected flocks/sheep. The farms were regularly visited by the farm vet (at least every third month), and lameness assessments were undertaken. The farmers performed detailed hoof checks of the whole flock (every hoof of every sheep) during routine hoof trimming.

For each group, 10 flocks were selected by convenience sampling. The selected flocks had an average size of 650 sheep (ranging 70 to 2800 sheep) of Merino Landschaf and Merino Landschaf mixed breeds.

As this was a field study, all participating animals stayed in the production chain after the trial and were not harmed.

### Sampling

The present study was double-blinded for grouping of the flock (sampling person) and the clinical outcome (laboratory). To detect *D nodosus* with a probability of more than 95% within a flock, the required sample size was calculated to be 30 for each flock (27 for one because of its smaller flock size, based on a PCR specificity for *aprV2* of 98% and a sensitivity of 90% [41].

The sampling took place in May and June 2018. All samples were processed and analysed in a single laboratory (Institute of Veterinary Bacteriology, University of Bern, Switzerland). Interdigital swabs were collected from all four feet of 30 sheep on each farm (27 on the smallest). In order to improve sensitivity, the flock was sampled according to a risk-based sampling plan [41]. High-risk animals were sampled first. These included clinically lame or recently purchased sheep, sheep which had been to shows or markets, and sheep with contact to other flocks. Thereafter, moderate-risk sheep were sampled: rams, sheep with claw health problems and sheep with poor horn quality. Then, “non-risk” sheep were randomly selected from the flock to gain the required sample size.

Each animal was identified by ear tag and sat down or put in a hoof trimming chute for sampling. The interdigital space was manually cleaned from soil or straw if necessary and scored according to the 0–5 footrot scoring system of the Swiss Consulting and Health Service for Small Ruminants [14], to assess the clinical free status.

For each sheep, new disposable gloves were worn to avoid contamination. A cotton swab (2 mm 15 cm, Paul Hartmann) was pulled through the interdigital space of the first claw, quarter-rotated and pulled through the second interdigital space, rotated again and proceeded like this until all four feet were sampled. The swabs were immediately placed for 1 min into a 96 deep-well plate (KingFisher™ 96 well clear round bottom 2 mL polypropylene deep well plate, Thermo Fisher Scientific) containing 1 mL SV–lysis buffer (4 M guanidinethiocyanate, 0.01 M Tris–HCl pH 7.5, 1% β-mercapto-ethanol) and afterwards discarded. For every flock, a new deep-well plate was prepared to prevent contamination between farms. Samples from the three pool-groups were separated on the plate for the same reason. The plates were sealed with a silicone cover (Thermo Fisher Scientific) and stored dark at 4 °C until laboratory analysis at 1 to 4 weeks after sampling. In total, 897 sheep from 30 different farms were sampled this way.

### Sample processing and real-time PCR

For every flock 50 μl aliquots from ten four-feet-samples (nine in the smallest flock) were pooled starting from high-risk animals resulting in three flock-samples with decreasing risk [41]. As a control, the VetMAX™ Xeno™ Internal Positive Control DNA (20.000 copies; Thermo Fisher Scientific) was added to each pooled sample prior to purification. The DNA was extracted as described [11], using a semi-automatic extraction robot (KingFisher™ Duo Prime, Thermo Fisher Scientific). The purified DNA samples were kept at −20 °C until PCR analysis.

The competitive real-time PCR [10] was performed in order to detect *aprV2* of virulent *D nodosus* and/or *aprB2* of benign *D nodosus* by use of the TaqMan™ Fast Advanced Master Mix and primer probe mix for the IPC (Thermo Fisher Scientific). If PCR-results of pools were positive for virulent *D nodosus*, the ten individual animal samples were PCR analysed individually to determine the number of positive animals. The same was done for pools with ambiguous results for the IPC.

## Data Availability

The datasets used and analysed during the current study are available from the corresponding author on reasonable request.

## Abbreviations

*aprB2*: gene coding for thermolabile acidic proteases
*aprV2*: gene coding for thermostable acidic proteases
Ct: Cycle threshold
*D nodosus*: *Dichelobacter nodosus*
IPC: Internal positive control
LAMP: Loop mediated isothermal amplification
PCR: Polymerase chain reaction
